# Volatile metabolome and floral transcriptome analyses reveal the volatile components of strongly fragrant progeny of *Malus* × *robusta*

**DOI:** 10.3389/fpls.2023.1065219

**Published:** 2023-01-20

**Authors:** Guofang Li, Jia Liu, He Zhang, Linguang Jia, Youxian Liu, Jiuyang Li, Shiwei Zhou, Pengjuan Wang, Ming Tan, Jianzhu Shao

**Affiliations:** ^1^ College of Horticulture, Hebei Agricultural University, Baoding, China; ^2^ Changli Institute of Pomology, Hebei Academy of Agricultural and Forestry Science, Changli, China

**Keywords:** floral fragrance, volatile components, linalool, methyl benzoate, leaf acetate, methyl anthranilate, transcriptome

## Abstract

Floral fragrance is an important trait that contributes to the ornamental properties and pollination of crabapple. However, research on the physiological and molecular biology of the floral volatile compounds of crabapple is rarely reported. In this study, metabolomic and transcriptomic analyses of the floral volatile compounds of standard *Malus robusta* flowers (Mr), and progeny with strongly and weakly fragrant flowers (SF and WF, respectively), were conducted. Fifty-six floral volatile compounds were detected in the plant materials, mainly comprising phenylpropane/benzene ring-type compounds, fatty acid derivatives, and terpene compounds. The volatile contents were significantly increased before the early flowering stage (ES), and the contents of SF flowers were twice those of WF and Mr flowers. Odor activity values were determined for known fragrant volatiles and 10–11 key fragrant volatiles were identified at the ES. The predominant fragrant volatiles were methyl benzoate, linalool, leaf acetate, and methyl anthranilate. In the petals, stamens, pistil, and calyx of SF flowers, 26 volatiles were detected at the ES, among which phenylpropane/benzene ring-type compounds were the main components accounting for more than 75% of the total volatile content. Functional analysis of transcriptome data revealed that the phenylpropanoid biosynthesis pathway was significantly enriched in SF flowers. By conducting combined analyses between volatiles and differentially expressed genes, transcripts of six floral scent-related genes were identified and were associated with the contents of the key fragrant volatiles, and other 23 genes were potentially correlated with the key volatile compounds. The results reveal possible mechanisms for the emission of strong fragrance by SF flowers, and provide a foundation for improvement of the floral fragrance and development of new crabapple cultivars.

## Introduction

*Malus robusta* have strong drought and stress resistance, grafting affinity, and high survivability, and thus has been widely used as a rootstock for apple production in China for many years. As a type of crabapple, the flowers develop readily from axillary buds. The flowers range in color from red to pink or pink and white, and have a light fragrance, and therefore are indicative of an excellent ornamental plant. The floral fragrance varies among individual plants, which reflects the type and quantity of volatiles emitted from the flowers. The fragrance characteristics are associated not only with the content of volatiles, but also the properties of the volatiles have a crucial effect on the floral fragrance. The key fragrant substances of the floral volatiles must have a high odor activity value, which is calculated from the actual concentration of the volatile substance compared with the threshold value of the substance (Saison et al., 2009b). In addition, the release of floral fragrance components is associated with different floral organs. For example, the petals and stamens contribute equally to the floral scent of *Ranunculus acris* (Bergström et al., 1995), and 70% of the essential oil of citrus flowers is produced in the stigma (Mactavish and Menary, 1997).

Several previous studies have extracted and identified floral volatile compounds released from apple flowers using slightly different methods. Using solvent extraction and gas chromatography–mass spectrometry (GC-MS), 70 volatiles were identified from 10 apple cultivars, including phenyl methanol and cinnamyl alcohol (Omata et al., 1990). However, no significant difference in chemical composition among the cultivars was detected. More than 40 volatiles detected by GC-MS were identified from the essential oil of apple flowers, such as limonene, geraniol, linalool, and benzenol (Buchbauer et al., 1993). In China, several studies have analyzed the volatile components of apple flowers but mainly focus on identification of the volatile species. The species and content of volatiles vary in different organs and developmental stages of apple flowers. Analysis of the volatiles of the bud, whole flower, petal, stamen, and calyx of Italian red meat apple ‘Pelingo’ revealed that linalool was the most abundant compound in the whole flower and various organs, and the second most abundant volatile was (E, E)-fariene (Fraternale et al., 2014).

Floral volatiles are secondary metabolites released from the flowers and generally are characterized by a low molecular weight, low boiling point, and high vaporization capability (Knudsen et al., 2006). More than 2000 types of floral volatiles are classified into seven categories, comprising terpenes, aliphatic compounds, phenylpropane/benzene ring-type compounds, carbon-containing 5-branch chain compounds, nitrogen compounds, sulfur-containing compounds, and other ring compounds (Dunkel et al., 2009; Bera et al., 2017). The majority of floral volatiles are concentrated in three categories: terpenes, aliphatic derivatives, and phenylpropane/benzene ring-type compounds (Rodriguez-Concepcion and Boronat, 2002; Pichersky and Dudareva, 2007; Muhlemann et al., 2014; Li et al., 2018). The synthesis pathways for phenylpropane/benzene ring compounds comprise an oxidative pathway dependent on coenzyme A, and a non-oxidative pathway independent of coenzyme A, or the combination of these two pathways (Pichersky and Gershenzon, 2002; Vogt, 2010; Muhlemann et al., 2014).

The development of functional genomics has enabled floral synthetic functional genes to be readily isolated and identified in non-model plants (Abbas et al., 2017; Gao et al., 2018). Transcriptomic sequencing has the advantages of high sensitivity, good repeatability, no need for technical replicates, and few starting samples. The structure and abundance of transcripts can be analyzed, and unknown transcripts and rare transcripts can also be detected to obtain more comprehensive transcriptomic information, which can facilitate further annotation and classification (Schuster, 2008). For example, analyses of the volatile components of gardenia flowers and leaves using GC-MS technology and transcriptome in full bloom enabled identification of many pathways and genes associated with the biosynthesis of floral volatiles, and revealed that the expression level of genes associated with floral metabolism was higher than that in leaves (Dhandapani et al., 2017). The highly expressed genes were enriched for the synthesis of esters, terpenes, and benzene ring compounds. An annotated petal expressed sequence tag database, in combination with metabolic and microarray expression analysis, has been used to compare differences in gene expression in the petals between scented and non-scented tetraploid rose cultivars, and detected 49 genes that were up-regulated in scented flowers. The detected genes were consistent with the release pattern of sesquiterpenes (Guterman et al., 2002). In rose, the expression patterns of these genes were determined by means of a transcriptome analysis, and it was speculated that the candidate genes *RrDXR* and *RrAAT* may be crucial regulators of terpenoid synthesis (Feng et al., 2014).

Plant floral fragrance plays a crucial role in attracting insects for pollination and can be used as a physical and mental health adjuvant treatment for humans by providing relaxation and a sense of pleasure. Improving floral fragrance in plants could not only enhance their commercial value but also ornamental value. Compared with flower color, flower type, and flowering period, floral fragrance is also an important quality trait of crabapple flowers, but is usually less highly valued as an ornamental property and for pollination. The flowers of most crabapple cultivars have a light fragrance and research on the floral scent volatiles is rarely reported. In the present study, the test material comprised novel germplasm with an unusually rich floral fragrance detected among progeny of *M. robusta*. The composition and volatile components of the floral fragrance were identified, and the floral transcriptome analyses at different stages of floral development were analyzed. These results provide a theoretical basis for the development and use of fragrant cultivars of crabapple.

## Materials and methods

### Materials and sampling

The study materials comprised 7-year-old *M. robusta* (Mr) trees and two individuals selected from among its progeny with strong and weak floral fragrance (SF and WF, respectively). The seedlings were planted in an apple nursery in Baoding, Hebei, China (38°55′N, 115°52′E);.

In early April 2019, branches bearing flowers at four developmental stages (early balloon stage, EB; late balloon stage, LB; early flowering stage, ES; and late flowering stage, LS) located outside the crown were collected and placed in beakers containing ultra-pure water, transported to the laboratory, and immediately placed in a growth chamber at 20°C with light intensity of 60 μmol·m^−2^·s^−1^ and photoperiod of 12 h light and 12 h dark. Collection and determination of volatiles from Mr, SF, and WF flowers were performed within 24 h after flower sampling.

The EB and LB flowers had unfolded, and closed petals. The ES flowers had opening petals and indehiscent stamens, and the LS flowers had fully opened petals and dehisced stamens. Images of floral stages were captured with a Canon 1500D in the macro mode.

Individual flowers at the EB, ES, and LS stages were sampled and stored at −80°C for RNA sequencing analysis. The sampled flowers were uniform at each stage.

### Measurement approach of floral volatiles

Ethyl decanoate was selected as the internal standard solution (IS) (Sigma, CAS# 110-38-3, Wang et al., 2019), and 2.59×10^−5^ mg solution was used as a test volatile component, which corresponded with the magnitude of the floral volatile contents, and was screened as a series diluted with methanol comprising 10^−3^, 10^−4^, 10^−5^, 10^−6^, and 10^−7^ dilution. Two complete flowers were selected, weighed, placed into a headspace sample bottle, and sealed. Next, IS was injected into the base of the bottle using a microsample inlet. Headspace–solid phase microextraction (HS-SPME) and GC-MS were performed to analyze the floral volatiles. Three biological replicates were performed for each sample.

The IS was loaded into the base of an inlet bottle and incubated upright in a constant temperature (50°C) water bath, incubated for 15 min, and then the extraction head slowly extend vertically into the inlet bottle to approximately 1 cm above the sample, and ceiling adsorption was allowed for 40 min. After adsorption, the extraction head was withdrawn and inserted into the inlet port of a gas chromatograph mass spectrometer (7890B-7000C, Agilent), and desorbed for 3 min at 250°C.

The GC conditions were as follows: capillary column HP-5MS (Agilent); high purity helium (99.999%) with a flow rate of 1.0 ml/min and inlet temperature of 250°C; the starting temperature was 50°C to 80°C and then increased at 5°C·min^-1^ to 240°C, and maintained for 1min at 240°C. The MS conditions were as follows: MS level IV rod temperature 260°C, ion trap temperature 230°C; ionization source EI, EI ionization energy 70 eV; and full scanning with mass range of 33-500 amu.

### Qualitative and quantitative analysis of floral volatiles

Based on the characteristic ion fragments and mass spectrum values, volatile components were identified by comparison with NIST 14 mass spectral library (Han et al., 2019; Wang et al., 2019; Fei et al., 2021). Quantitative analysis was based on the peak area of ethyl decanoate, which was added to each sample as an internal standard. Using qualitative analysis software to calculate the peak areas of identified volatiles, a statistical analysis of the trial data was performed using Excel 2019 and SPSS software. The results of the measured data were represented by the mean value. The relative area of each component was calculated using the peak area normalization method with the internal solution. The sum of the identified volatiles was taken to be the total volatile content.

The odor activity values (OVAs) were calculated as the volatile component concentration divided by the volatile threshold. Those OVAs greater than 1.0 were determined to be the key fragrant volatiles. The odor thresholds were obtained from the relevant literature (Pino and Quijano, 2012; Zhu et al., 2018; Kang et al., 2019).

Following the above procedures, the floral volatiles in the petals, stamens, pistil, and calyx and bracts of SF flowers were analyzed, with three biological replicates for each floral part.

### Transcriptome sequencing of the whole flowers and data analysis

RNA-sequencing analyses were performed using flowers at the EB, ES, and LS stages. Each sample was repeated in biological replicates, and the sequencing was conducted on an Illumina HiSeq X Ten system by Novogene (Beijing, China). The sequencing steps comprised standard extraction and quality control of RNA samples, library construction and quality assessment, Illumina sequencing of the 150 bp paired-end reads, and sequence mapping to the apple HFTH1 Whole Genome v1.0.

The gene expression level was calculated using the fragments per kilobase of exon per million mapped fragments (FPKM) value. Differential expression analysis between two comparison combinations was performed using the DESeq2 R package, and the resulting P-values were adjusted to control the false discovery rate. The |log_2_(FoldChange)|> 0 and padj <0.05 were used as the criteria for screening differentially expressed genes (DEGs). Identification of DEGs, gene ontology (GO) analysis, and Kyoto Encyclopedia of Genes and Genomes (KEGG) pathway enrichment analysis were performed with cluster Profiler, and *p*_adj_ *<*0.05 was used as the threshold for significant enrichment. Combined analyses of DEG functions and expression levels were conducted with MapMan3.6.0RC1, which is a user-driven tool that visualizes large datasets as diagrams of metabolic pathways or other processes. The Mdomestica_196 library was used for mapping.

### Connection analyses among volatiles and DEGs

To construct correlation networks, Pearson correlation coefficients (PCCs) were calculated between the volatile components and DEGs. In this study, a |PCC value| ≥ 0.7 was considered to indicate a correlation. For all pairs with a significant PCC value and *P*-value ≤ 0.05, we also calculated mutual rank values ≤ 10 between pairs to determine a significant connection (Obayashi and Kinoshita, 2009; Aya et al., 2011). These calculations were performed using R/Bioconductor. The networks were visualized using Cytoscape software v3.7.2.

## Results

### Flower phenotypes and volatile compound contents

**Figure 1:**
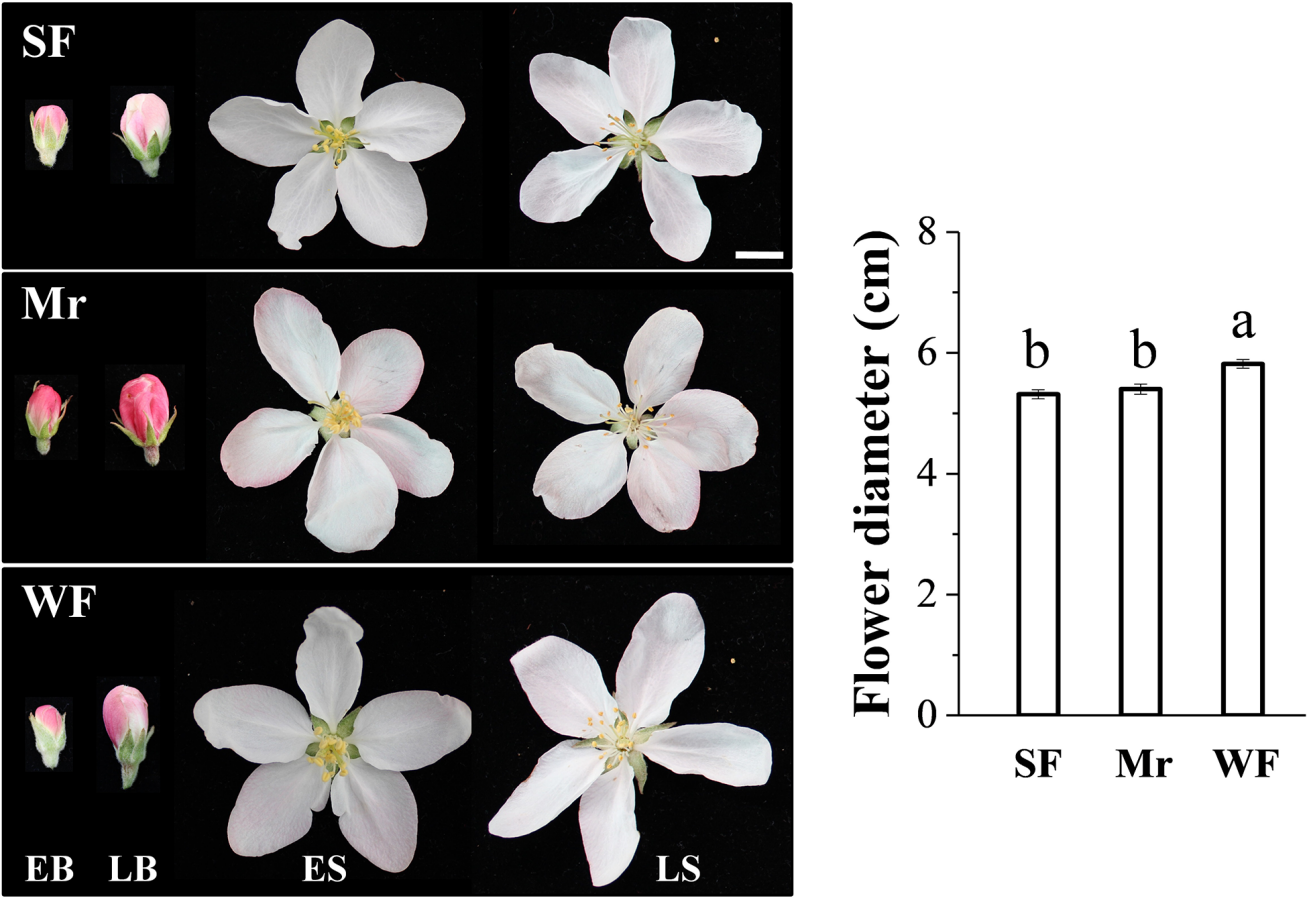
Flower phenotypes at four developmental stages in the *Malus robusta* materials. EB, early balloon stage; LB, late balloon stage; ES, early flowering stage; LS, late flowering stage; Mr, standard *M. robusta*; SF, strongly fragrant progeny; WF, weakly fragrant progeny. The histogram shows the flower diameter of each material at the LS stage. Different lowercase letters indicate a significant difference using one-way ANOVA followed by Tukey’s test (*P* < 0.05). Scale bars = 10.0 mm.

The diameters of Mr and SF flowers were similar, which were significantly smaller than that of WF flowers (**Figure 1**). The petals of Mr, WF, and SF flowers were distinctly pink at EB and LB; the color was more obvious in Mr flowers, and the color of WF and SF flowers was similar (**Figure 1**). The petals of Mr and WF flowers were slightly pink, whereas SF petals were essentially white, at ES and LS. No distinct differences in the phenotypes of the stamens, pistil, calyx, and bracts of SF and WF flowers were observed (**Figure S1**).

**Figure 2:**
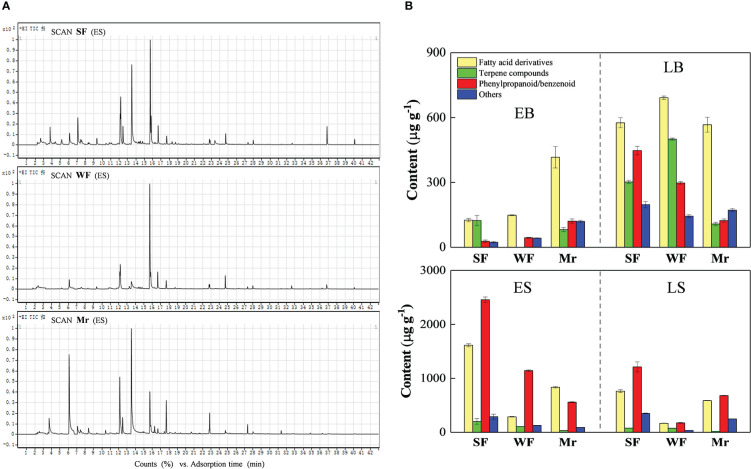
Chromatograms and composition of floral scent volatiles in the *Malus robusta* materials. **(A)**, Chromatograms for strongly fragrant (SF), standard (Mr), and weakly fragrant (WF) flowers at the early flowering stage. **(B)**, Composition and species number of volatiles at four stages of floral development. Data represent the means (n= 3).

The chromatograms for Mr, SF, and WF flowers were used to identify volatile components. Total ion maps for Mr, SF, and WF flowers at ES were shown in **Figure 2A**. A total of 56 volatile components in Mr, SF, and WF flowers were identified in total (**Table S1**). The volatile species were mainly phenylpropane/benzene ring-type compounds, fatty acid derivatives, and terpene compounds (**Figure 2B** and **Tables S1-S4**).

Total volatile contents of Mr, SF, and WF flowers initially increased at EB, LB, and ES, and thereafter decreased at LS, except that similar contents between ES and LS were observed for Mr (**Figure 2B**). The highest volatile contents in Mr, SF, and WF flowers were observed at ES. Among the three materials at ES, the total volatile content of SF flowers was 4563.15 µg·g^-1^, which was 2.7 times and 3.0 times higher than those of WF (1668.14 µg·g^-1^) and Mr (1516.55 µg·g^-1^), respectively. In SF flowers, the number of volatile species detected was 19, 20, 20 and 19 at EB, LB, ES, and LS, respectively (**Tables S2-S4**). In comparison, at these stages, the number of volatile species detected was 12, 15, 18, and 16 for WF, and 16, 18, 23, and 20 for Mr, respectively (**Tables S2-S4**). These results indicated that ES was the main stage for volatiles emission in Mr, SF, and WF flowers.

### Characteristics of the floral volatiles at the ES stage

At ES, the most abundant volatile compounds in SF flowers were phenylpropane/benzene ring-type compounds, reaching 53.88% of the total content (4563.15 µg·g^-1^) (**Table 1**). Among these compounds, the content of methyl benzoate (v06) was highest, followed by benzyl alcohol (v25), leaf acetate (v10), and methyl anthranilate (v26). In WF, the most abundant compounds were also phenylpropane/benzene ring-type compounds, reaching 68.56% of the total content (**Table 1**). The volatiles with the highest contents were methyl benzoate (v06), benzyl alcohol (v25), and benzaldehyde (v28). In Mr, the predominant volatiles were phenylpropane/benzene ring-type compounds and fatty acid derivatives, and the volatiles with the highest contents were methyl benzoate (v06) and leaf acetate (v10), reaching 47.96% and 38.97% of the total content, respectively (**Table 1**).

**Table 1:** Contents of floral volatiles at the ES stage.

Volatile Name	Code	Content (µg·g^-1^)		
		SF	WF	Mr
Methyl benzoate	v06	692.90 ± 5.72a	476.46 ± 5.08b	411.88 ± 7.11c
Methyl laurate	v02	65.82 ± 4.81a	53.25 ± 1.44b	21.27 ± 0.79c
Methyl hexanoate	v22	56.35 ± 4.96a	11.99 ± 0.90b	55.13 ± 1.18a
(E)-3-Hexen-1-ol	v07	394.70 ± 7.84a	54.97 ± 0.84c	154.38 ± 3.88b
1,3-Dimethylbenzene	v13	384.79 ± 9.43a	61.93 ± 2.50b	18.54 ± 1.42c
Decane	v08	233.12 ± 45.38a	83.93 ± 4.58b	73.48 ± 2.36b
Linalool	v01	201.16 ± 52.52a	40.72 ± 0.26b	32.12 ± 0.59b
Benzaldehyde	v28	193.48 ± 22.90a	218.99 ± 6.67a	8.51 ± 1.04b
Naphthalene	v12	128.96 ± 4.71a	6.19 ± 0.11b	9.74 ± 0.08b
Methyl octanoate	v02	128.88 ± 28.29a	60.30 ± 0.42b	–
Leaf acetate	v10	419.96 ± 6.05a	–	217.17 ± 10.38b
Methyl hexadecanoate	v04	80.87 ± 4.93a	17.78 ± 2.74b	–
Benzyl alcohol	v25	668.47 ± 15.69a	243.03 ± 8.28b	–
Methyl 2-methylbutyrate	v16	48.48 ± 6.02a	–	17.42 ± 0.59b
Methyl anthranilate	v26	390.20 ± 2.14	–	–
Hexyl alcohol	v27	379.57 ± 3.70	–	–
Methyl acetate	v21	39.19 ± 4.47	–	–
Propylcyclopropane	v29	31.53 ± 0.81	–	–
2-Methyldecane	v30	14.27 ± 0.28	–	–
4-Methyl-Decanes	v18	10.45 ± 0.40	–	–
1-Phenylpropane-1,2-diol	v40	–	92.60 ± 1.30	–
Linalyl acetate	v05	–	68.92 ± 1.36	–
2-Ethylhexanol	v23	–	49.69 ± 2.59	–
Indole	v41	–	44.47 ± 4.28	–
Tetracosane	v42	–	43.76 ± 4.28	–
Methyl tetradecanoate	v09	–	39.19 ± 1.00	–
Methyl 2-Ethylhexanoate	v47	–	–	91.57 ± 1.66
cis-3-Hexenyl formate	v20	–	–	89.10 ± 2.48
4-Methoxy phenyl oxime	v31	–	–	78.56 ± 1.98
Cyclohexanol	v44	–	–	64.96 ± 0.18
Methyl 2-methyl-2-butenoate	v48	–	–	52.57 ± 1.95
Hexyl acetate	v49	–	–	31.31 ± 0.20
Benzyl acetate	v50	–	–	31.14 ± 0.23
Methyl heptenone	v35	–	–	29.03 ± 0.17
Acethydrazide	v51	–	–	11.41 ± 1.14
2-Methylgeng acid	v52	–	–	8.09 ± 0.13
2-Methylcyclopentalol	v53	–	–	5.24 ± 0.31
Methyl butyrate	v54	–	–	3.91 ± 0.54

Values were means ± SE. Lowercase letters indicate significant differences determined using one-way ANOVA followed by Tukey’s test ANOVA analysis (P < 0.5). ‘-’ represents that the substance was not detected.

Analysis of the floral volatiles in Mr, SF, and WF flowers at ES revealed that 9 volatiles were detected in the three flowers, of which 7 volatiles were significantly more abundant in SF (**Table 1**). Five volatiles detected in SF flowers were in common with WF and Mr, and the contents of the 5 volatiles were significantly higher in SF. In SF flowers, 6 volatiles were unique, of which those with higher contents in SF were methyl anthranilate (v26) and hexyl alcohol (v27). In contrast, 6 and 12 volatiles were detected only in WF and Mr, respectively.

### Identification of the key and predominant floral volatiles at the ES stage

Applying the relevant volatile threshold (**Table S5**), the OVAs for each volatile were calculated and summarized at ES (**Table 2**). For SF and Mr, 11 key floral fragrant volatiles were identified. Compared with WF and Mr, the OVAs of four volatiles comprising methyl benzoate (v06), linalool (v01), leaf acetate (v10), and methyl anthranilate (v26) were significantly higher in SF and all were greater than 30000. The OVAs of methyl benzoate (v06), linalool (v01), leaf acetate (v10), hexyl acetate (v49), and benzyl acetate (v50) were higher and greater than 5000 in Mr. In WF, two OVAs among the 10 key floral volatiles identified, methyl benzoate (v06) and linalool (v01), were higher and greater than 5000.

**Table 2:** Odor activity values of key floral volatiles.

Volatile Name	Code	SF	WF	Mr
Methyl benzoate	v06	1.33×10^6^ ± 1.10×10^4^a	9.15×10^5^ ± 9.76×10^3^b	7.92×10^5^ ± 1.37×10^4^c
Methyl hexanoate	v22	670.82~ ± 59.08a	142.74 ± 10.71b	656.35 ± 14.06a
		~804.98 ± 70.89a	~171.29 ± 12.85b	~787.62 ± 16.87a
(3E)-3-Hexen-1-ol	v07	254.65 ± 5.06a	35.46 ± 0.54c	99.60 ± 2.50b
1,3-Dimethylbenzene	v13	69.96 ± 1.71a	11.26 ± 0.45b	3.37 ± 0.26c
Linalool	v01	3.35×10^4^ ± 8.75×10^3^a	6.78×10^3^ ± 43.81b	5.35×10^3^ ± 98.42b
Benzaldehyde	v28	55.28 ± 6.54a	62.57 ± 1.90a	2.43 ± 0.30b
		~552.81 ± 65.42a	~625.67 ± 19.04a	~24.31 ± 2.97b
Methyl octanoate	v02	644.39 ± 141.47a	301.48 ± 2.09b	–
Leaf acetate	v10	3.47×10^4^ ± 499.82a	–	1.79×10^4^ ± 857.69b
Benzyl alcohol	v25	66.85 ± 1.57a	24.30 ± 0.83b	–
Methyl anthranilate	v26	1.30×10^5^ ± 714.67	–	–
Hexyl alcohol	v27	151.83 ± 1.48	–	–
Linalyl acetate	v05	–	68.92 ± 1.36	–
		–	~689.17 ± 13.64	–
Indole	v41	–	317.65 ± 30.59	–
Hexyl acetate	v49	–	–	1.56×10^4^ ± 100.87
Benzyl acetate	v50	–	–	1.56×10^4^ ± 114.09
Methyl heptenone	v35	–	–	580.64 ± 3.50
Methyl butyrate	v54	–	–	51.49 ± 7.09
		–	–	~65.22 ± 8.98

Values were means ± SE. Lowercase letters indicate significant differences determined using one-way ANOVA followed by Tukey’s test ANOVA analysis (P < 0.5). ‘-’ represents that the substance was not detected.

The OVAs of methyl benzoate (v06), linalool (v01), and leaf acetate (v10) in SF were significantly higher than those in WF and Mr, which was consistent with the difference in volatile contents (**Table 1**). In particular, methyl anthranilate (v26), a floral volatile unique to SF, had a high OVA of 1.30×10^5^. Therefore, the high contents and OVAs of common and unique volatiles were indicated to be the primary reasons for the strong fragrance in SF flowers. In addition, methyl benzoate (v06), linalool (v01), leaf acetate (v10), and methyl anthranilate (v26) were indicated to be the predominant fragrant volatiles in SF flowers at ES.

### Volatiles in different organs of SF flowers at ES

In total, 26 volatile compounds were identified in the petals, stamens, pistil, and calyx bracts of SF flowers (**Figure S1** and **Table 3**). Based on the relative contents of the volatile compounds, phenylpropane/benzene ring-type compounds were the predominant volatile compounds in the four parts (78.71%, 83.17%, 96.01% and 88.31%, respectively). In petals, 11 volatile compounds were detected, among which those with relatively high contents were benzyl alcohol (v25; 51.92%), methyl anthranilate (v26; 13.46%), and leaf acetate (v10; 11.84%). 2-Ethylhexanol (v23), benzyl butyrate (v66), and naphthalene (v12) were only detected in the petals. In the stamens, 18 volatiles were detected, among which those with the highest contents were benzyl alcohol (v25; 47.00%) and methyl anthranilate (v26; 17.94%). The volatile compounds detected only in stamens were 3-aminophenylacetylene (v38), benzyl benzoate (v65), and heneicosane (v11). In the pistil, 7 volatile compounds were detected, among which those with the highest contents were benzyl alcohol (v25) and methyl anthranilate (v26). The only compound detected solely in the pistil was *cis*-3-hexenyl formate (v20). In the calyx and bracts, 18 volatile compounds were detected. The relative contents of benzyl alcohol (v25; 41.30%) and methyl anthranilate (v26; 23.57%) were highest. Linalyl butyrate (v34), indole (v41), geranylacetone (v58), and *cis*-3-hexenyl benzoate (v59) were only identified in the calyx and bracts. Thus, both benzyl alcohol (v25) and methyl anthranilate (v26) were detected in all floral organs and their relative contents were the highest among all volatile compounds detected.

**Table 3:** Relative contents of volatiles in different floral organs (%).

Volatile Name	Code	Petal	Stamen	Pistil	Calyx and bracts
Benzyl alcohol*	v25	51.92 ± 0.72	47.00 ± 0.84	50.91 ± 2.52	41.30 ± 1.12
Methyl anthranilate*	v26	13.46 ± 1.68	17.94 ± 0.53	26.80 ± 3.38	23.57 ± 1.14
Indole*	v41	–	–	–	9.95 ± 0.30
Cinnamyl alcohol*	v57	9.86 ± 0.45	5.50 ± 0.37	–	6.12 ± 0.41
(E)-3-Hexen-1-ol	v07	4.30 ± 0.44	4.74 ± 0.34	–	4.85 ± 0.22
Leaf acetate	v10	11.84 ± 1.18	4.69 ± 0.22	–	2.79 ± 0.16
Linalyl butyrate	v34	–	–	–	1.78 ± 0.07
Linalool	v01	–	4.58 ± 0.45	–	1.44 ± 0.07
Benzyl acetate*	v50	1.64 ± 0.11	2.10 ± 0.18	–	1.29 ± 0.04
Methyl benzoate*	v06	0.27 ± 0.01	2.06 ± 0.16	6.01 ± 0.77	1.27 ± 0.02
Geranylacetone	v58	–	–	–	0.82 ± 0.07
Benzaldehyde*	v28	–	2.44 ± 0.11	6.37 ± 0.21	0.81 ± 0.03
cis-3-Hexenyl benzoate*	v59	–	–	–	0.81 ± 0.03
Phenethyl alcohol*	v60	–	1.15 ± 0.04	3.36 ± 0.42	0.81 ± 0.03
3-Phenyl-1-propanol*	v61	–	0.75 ± 0.04	–	0.70 ± 0.01
Cinnamaldehyde*	v62	–	1.05 ± 0.13	–	0.61 ± 0.07
Benzyl 2-methyl-2-butenoate*	v63	–	0.53 ± 0.03	2.56 ± 1.02	0.58 ± 0.05
Pentanoic acid*	v64	–	0.48 ± 0.02	–	0.49 ± 0.06
2-Ethylhexanol	v23	3.96 ± 0.15	–	–	–
3-Aminophenylacetylene	v38	–	1.63 ± 0.81	–	–
Benzyl benzoate*	v65	–	0.54 ± 0.04	–	–
Benzyl butyrate*	v66	0.84 ± 0.09	–	–	–
Heneicosane	v11	–	0.94 ± 0.03	–	–
cis-3-Hexenyl formate	v20	–	–	3.98 ± 0.19	–
Naphthalene*	v12	0.72 ± 0.08	–	–	–
Hexyl alcohol	v27	1.18 ± 0.34	1.88 ± 0.12	–	–

Values were means ± SE. ‘*’ represents the phenylpropane/benzene ring-type compound. ‘-’ represents that the substance was not detected.

The four compounds methyl benzoate (v06), linalool (v01), leaf acetate (v10), and methyl anthranilate (v26), which contributed to the strong fragrance of SF flowers, were detected in the stamens, calyx, and bracts. The relative contents of methyl anthranilate (v26; 26.80%) and methyl benzoate (v06; 6.01%) were highest in the pistil. The relative content of linalool (v01; 4.58%) was highest in the stamens. The relative content of leaf acetate (v10; 11.84%) was highest in the petals. These results indicated that emission of the volatile species and the contents of floral volatiles varied between intact flowers and different floral organs.

### Analysis of DEGs from the transcriptome data

**Figure 3:**
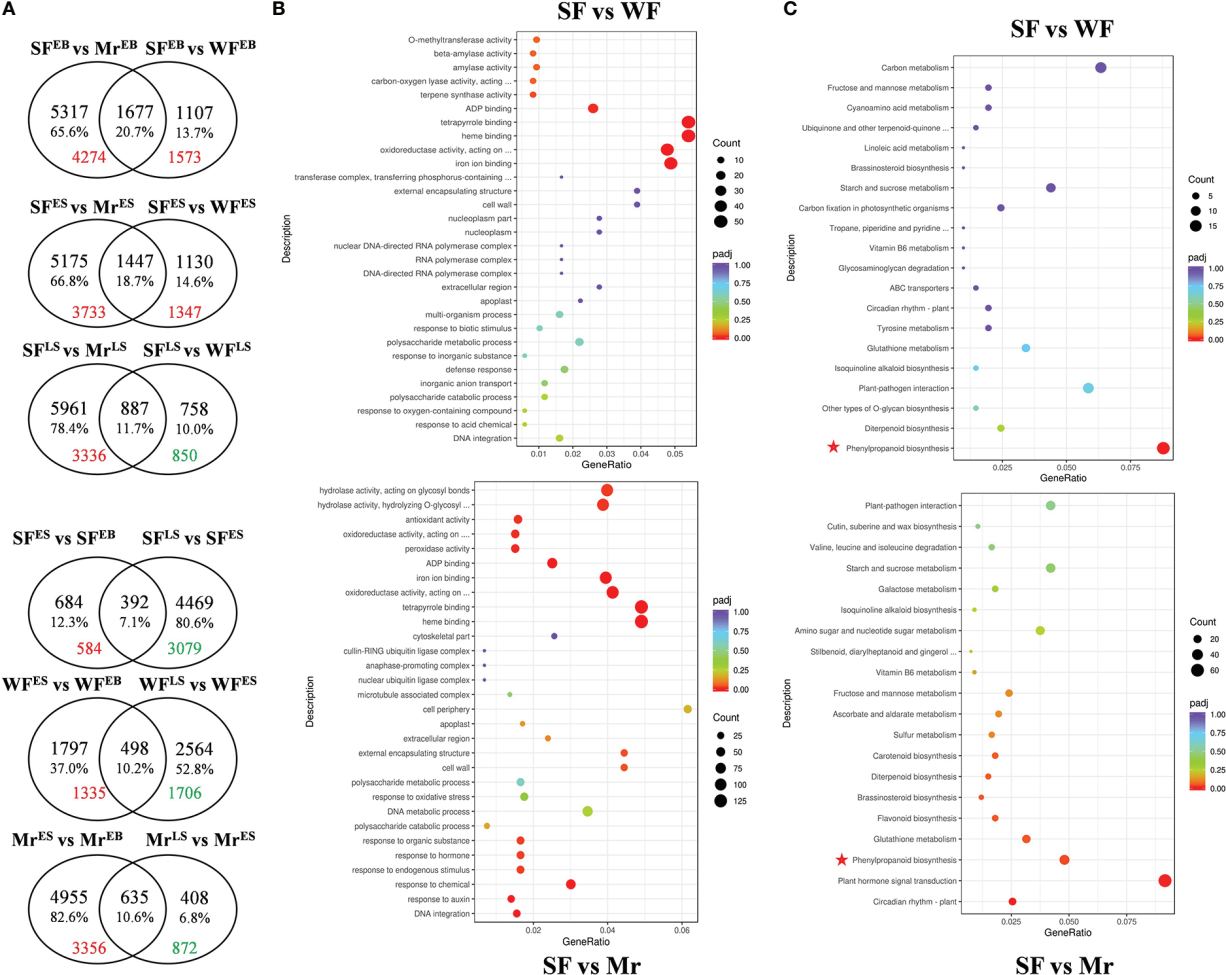
Venn diagram, GO terms, and KEGG pathways for differentially expressed genes (DEGs). **(A)** Venn diagram of genes expressed in the different plant materials and floral development stages. The intersections indicate the number of genes in common. Red and green numbers indicate the number of up- and down-regulated DEGs, respectively, in the corresponding pair. **(B, C)** GO terms and KEGG pathways for comparisons of samples (SF *vs* WF and SF *vs* Mr) at the ES stage. Different colors for *p*_adj_ indicate the significance level, and the count size indicates the number of DEGs. Red asterisks indicate the common pathway in the comparison of SF with WF and Mr.

There 6.34 ~ 11.36 Gb clean Illumina reads were retained from every sample of SF, WF, and Mr flowers (**Table S6**). The percentages of bases with Q20 and Q30 were 97.93 ~ 98.08% and 93.58 ~ 94.41%, respectively, and the error rates were < 0.03. Based on the high quality of transcriptome data and screening criteria, comparisons of DEGs in SF, WF, and Mr flowers were examined at EB, ES, and LS. In SF flowers, 1076 and 4861 DEGs were detected in the comparison between ES and EB, and between LS and ES, respectively, among which 584 were up-regulated between ES and EB, and 3079 were down-regulated between LS and ES (**Figure 3A**). In WF flowers, 2295 and 3062 DEGs were detected in the comparison between ES and EB, and between LS and ES, respectively. In Mr flowers, 5590 and 1043 DEGs were detected in the comparison between ES and EB, and between LS and ES, respectively. In the same materials, generally, a greater number of DEGs were up-regulated at ES than that at EB or LS. From the comparisons between the same stage, 1573 and 1347 DEGs at EB and ES were up-regulated, respectively, whereas 850 DEGs at LS were down-regulated. In the comparison of SF and Mr, a greater number of DEGs were up-regulated at EB, ES, and LS (4274, 3733, and 3336, respectively).

### Annotation of DEGs with the GO and KEGG databases

Based on the differences in content of the volatiles, the GO and KEGG analyses were used to annotate the DEGs among SF, Mr, and WF at ES and LS (**Figures 3B, C** and **Table S7**). At ES, 802 GO terms were obtained for the comparison between SF and WF. Six terms in the molecular function (MF) category were significantly enriched, which were associated with binding of Iron ion (GO:0005506), Heme (GO:0020037), Tetrapyrrole (GO:0046906), and ADP (GO:0043531), activity of Terpene synthase (GO:0010333), and Oxidoreductase (GO:0016705). In the comparison of SF and Mr, 1119 terms were obtained, of which 20 terms in the biological process (BP), MF, and cellular component (CC) categories were significantly enriched. These enriched terms were associated with DNA integration, response to Auxin (GO:0009733), Chemical (GO:0042221), Endogenous stimulus (GO:0009719), Hormone (GO:0009725), and Organic substance (GO:0010033) in the BP category, and Cell wall (GO:0005618) and External encapsulating structure (GO:0030312) in the CC category, and binding of Heme (GO:0020037), Tetrapyrrole (GO:0046906), Iron (GO:0005506), and ADP (GO:0043531), activity of Oxidoreductase (GO:0016705, GO:0016684, and GO:0016682), Peroxidase (GO:0004601), Antioxidant (GO:0016209), Hydrolase (GO:0004553 and GO:0016798), and Beta-amylase (GO:0016161) in the MF category. Compared with WF, 85 KEGG pathways were obtained, of which the phenylpropanoid biosynthesis pathway (mdm00940) was significantly enriched among the DEGs that were upregulated in SF. In the comparison of SF with Mr, 109 KEGG pathways were obtained, of which Circadian rhythm–plant (mdm04712), Plant hormone signal transduction (mdm04075), Glutathione metabolism (mdm00480), and biosynthesis of Phenylpropanoid (mdm00940), Flavonoid (mdm00941), Brassinosteroid (mdm00905), and Diterpenoid (mdm00904) were significantly enriched. Importantly, the pathway of Phenylpropanoid biosynthesis was significantly identified in the comparison of SF with WF and Mr.

At LS, 690 GO terms were obtained in the comparison between SF and WF, of which the MF term ADP binding (GO:0043531) was significantly enriched. In the comparison of SF with Mr, 1088 terms were obtained of which 16 MF terms were significantly enriched. These enriched terms were associated with activity of Oxidoreductase (GO:0016705, GO:0016701, GO:0016682, GO:0016702 and GO:0016679), Dioxygenase (GO:0051213), Amylase (GO:0016160), Drug transmembrane transporter (GO:0015238), Beta-amylase (GO:0016161), and Transferase (GO:0016758), binding of ADP (GO:0043531), Sequence-specific DNA (GO:0043565), Iron ion (GO:0005506), Heme (GO:0016758), Tetrapyrrole (GO:0046906), and Coenzyme (GO:0016758). In the comparison with WF, 79 KEGG pathways were obtained in SF. In the comparison of SF with Mr, 111 KEGG pathways were obtained of which Phenylpropanoid biosynthesis (mdm00940), Valine, leucine and isoleucine degradation (mdm00280), Carotenoid (mdm00906) and Brassinosteroid (mdm00905) biosynthesis, and metabolism of Glutathione (mdm00480), Ascorbate and aldarate (mdm00053), Cyanoamino acid (mdm00460), and Starch and sucrose (mdm00500) were significantly enriched.

**Figure 4:**
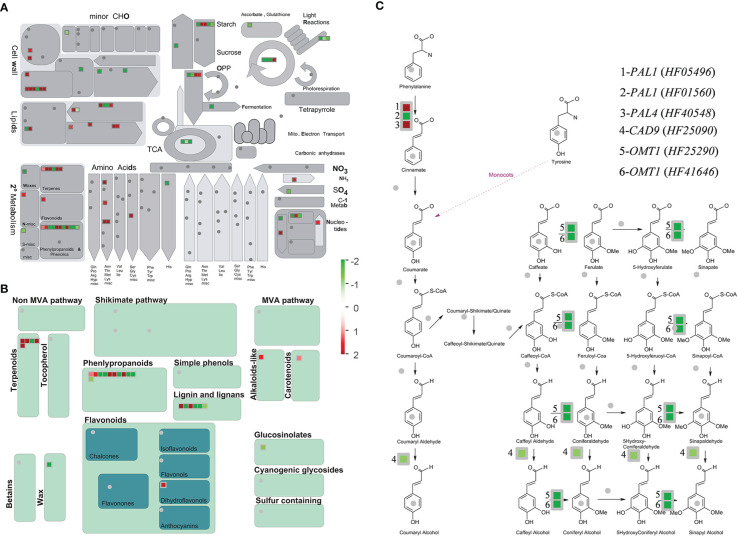
Metabolism overview and secondary metabolism of the identified DEGs associated with metabolic pathways for floral volatiles. **(A, B)** MapMan was used to classify the DEGs from the comparisons SF *vs* (WF and Mr). **(C)** Mapped DEGs involved in the phenylpropanoid pathway. Blue and red colors represent up- and down-regulated genes, respectively. Values represent the fold difference in expression level. Each square represents a DEG. Bins (or sub-bins) are shown as a block, which represent different metabolic pathways.

From visual analysis of the DEGs with MapMan (**Figure 4A, B**), 82 metabolism-related genes were listed in the comparisons between SF and WF and Mr at ES, among which 27 secondary metabolite-related genes were listed. The greatest number of DEGs involved in secondary metabolism were associated with phenylpropanoids, of which 11 genes were detected at ES (**Figure 4B**). In addition, DEGs involved in secondary metabolism were associated with terpenoids, of which 5 genes were detected at ES. In the phenylpropanoid pathway, *MrPAL1*, *MrPAL4*, *MrCAD9*, and *MrOMT1* genes were significantly mapped (**Figure 4C**), which may indicate that these genes played important roles in the strong emission of floral fragrance by SF flowers at ES.

### Pathways and genes correlated with production of predominant floral volatiles

Combined analyses of the identified KEGG pathways and the predominant floral volatiles revealed that the key pathways were associated with the synthesis of methyl benzoate, methyl anthranilate, linalool, and leaf acetate. As a result of KEGG mapping to the apple genome, phenylalanine metabolism (mdm00360), phenylalanine, tyrosine and tryptophan biosynthesis (mdm00400) and biosynthesis of amino acids (mdm01230), terpenoid backbone biosynthesis (mdm00900), and α-Linolenic acid metabolism (mdm00592) were associated with the synthesis of methyl benzoate, methyl anthranilate, linalool, and leaf acetate, respectively.

Based on previous studies, homologous genes associated with production of floral volatiles were screened in apple. Eighteen, 3, 17, and 5 of the 43 genes were identified in the synthesis pathways for linalool (v01), methyl benzoate (v06), leaf acetate (v10), and methyl anthranilate (v26), respectively (**Table S8** and **Figure 6A**). As revealed by the transcriptome data, *HF24990* (*PAL*) and *HF42215* (*BAMT*), and *HF43372* (*AS*) were closely associated with methyl benzoate (v06) and methyl anthranilate (v26) synthesis, respectively. *HF16930* and *HF18003* (*LOX*), and *HF10162* (*AAT*) were closely associated with the synthesis of leaf acetate (v10). Non-significant correlations were observed between the transcripts of more homologous genes and the corresponding volatiles, such as linalool (v01), which may be one reason that the syntheses of floral volatiles were regulated by multiple genes, or species variability in apple.

### Connection analyses between floral volatiles and DEGs

**Figure 5:**
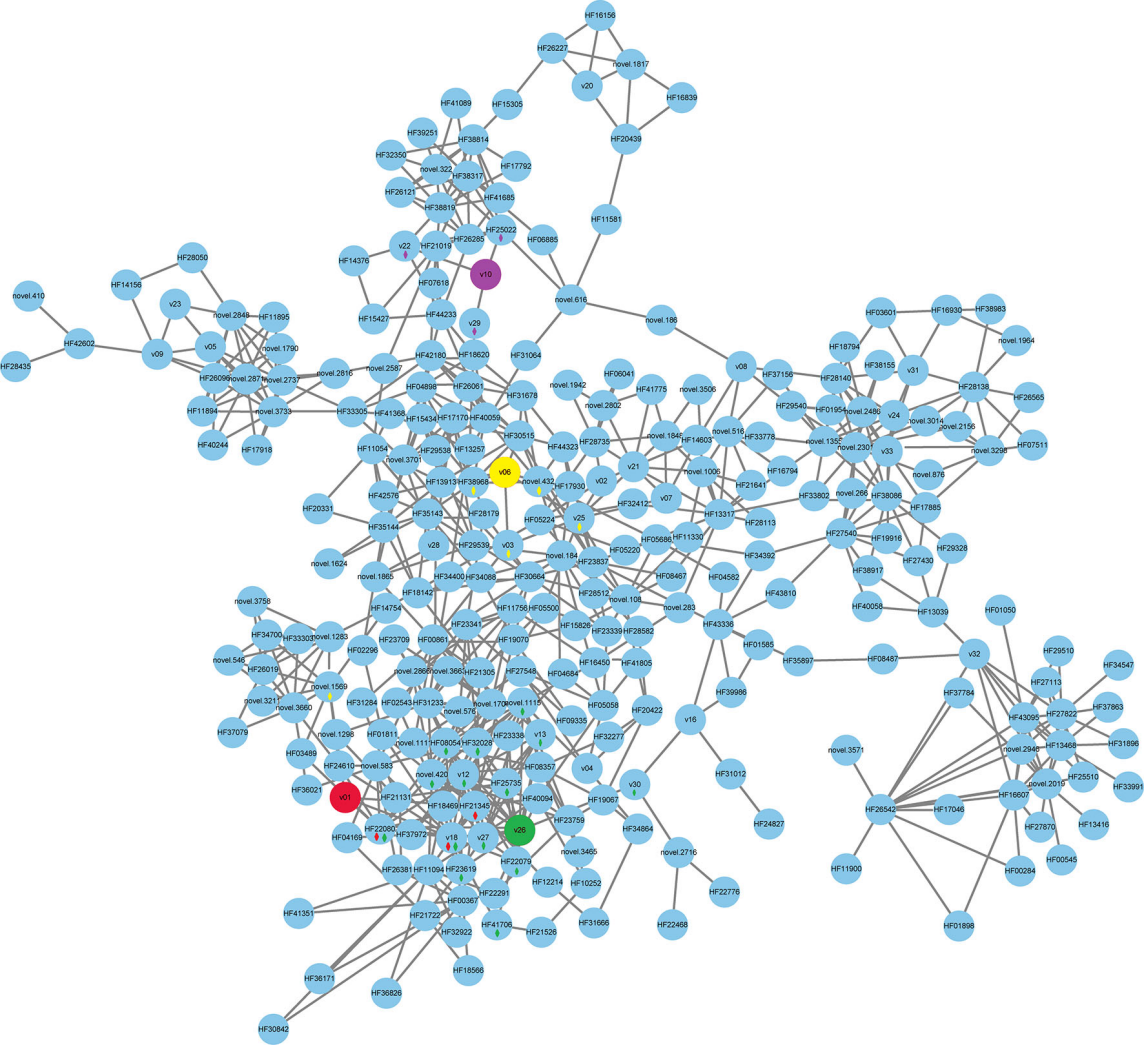
Correlation network for volatiles and DEGs. A correlation subnetwork was constructed using the data for DEGs shared by pairs of samples in the Venn diagrams and the content of the floral volatiles. The 4 fragrant volatiles, comprising linalool (leaf acetate, v01, color red), methyl benzoate (v06, color yellow), leaf acetate (v10, color purple), and methyl anthranilate (v26, color green), were used as guide data and are labeled with different colors. A correlation between v01, v06, v10, v26 and DEGs or other volatiles was labeled by a diamond with the corresponding color.

**Figure 6:**
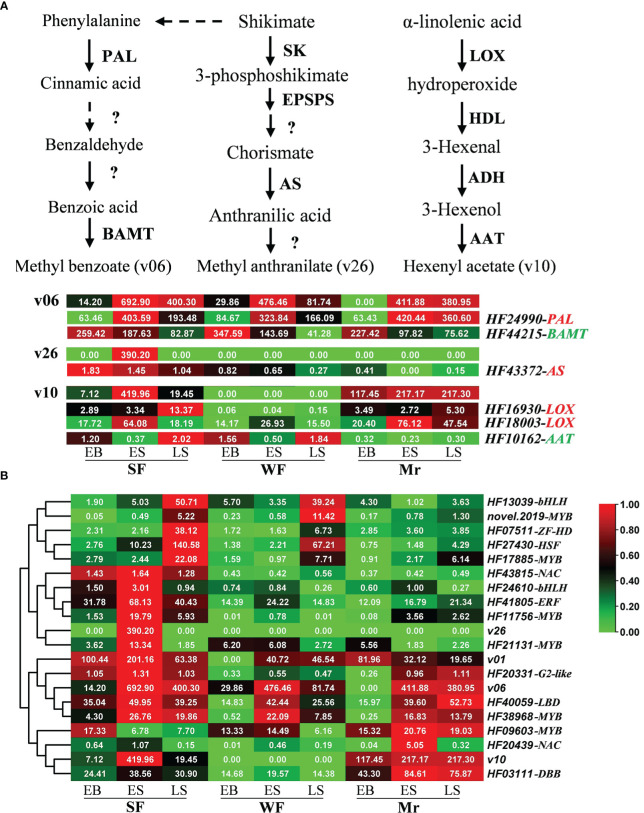
Expression patterns of genes associated with floral volatiles. **(A)** Expression patterns of homologous genes corresponding to the synthetic pathway of methyl benzoate (v06), hexenyl acetate (leaf acetate, v10), and methyl anthranilate (v26) were analyzed at the different developmental stages of strongly fragrant (SF), standard (Mr), and weakly fragrant (WF) flowers. Continuous arrows depict a one-step enzymatic reaction. **(B)** Identification of transcription factors potentially associated with floral volatiles based on the correlation network. In the heatmap, red and green colors represent the maximum and minimum values, respectively, and each line is independent. The first list on the right is the volatile ID and gene ID, and the second list is the gene name. The red and green backgrounds behind the gene name indicate a positive and negative correlation, respectively. Data represent the means (n= 3).

Connection and heatmap analyses between the floral volatiles and quantified DEGs were performed. The volatile contents of linalool (v01), methyl benzoate (v06), leaf acetate (v10), and methyl anthranilate (v26) were reference cores (**Figures 5**, **6**). As shown in **Figure 5** and **Table S9**, v18, and *HF21345*, *HF22080*, and *novel.1569* were positive correlated with the content of linalool (v01). Volatiles and DEGs positively correlated with methyl benzoate (v06) were v03, v25, and *HF38968*, and *novel.432*. Volatiles and DEGs positively correlated with leaf acetate (v10) were v22 and v29, and *HF25022*. V30, v13, v12, v27, v18, *HF32028*, *HF25735*, *novel.420*, *novel.1115*, *HF22079*, *HF41706*, *HF22080*, *HF08054*, and *HF23619* were correlated with methyl anthranilate (v26). These DEGs were correlated with cupredoxin (*HF21345*), terpene synthase (*HF22080*), myb domain protein (*HF38968*), peroxidase (*HF25735* and *HF23619*), Terpenoid cyclases/Protein prenyltransferases (*HF22079*), Nucleic acid-binding (*HF41706*), cytochrome P450 (*HF08054*), and some unknown functions (**Table S9**).

In further analysis, 80 DEGs among the comparisons of SF with WF or Mr were identified as transcription factors and were mainly associated with the MYB (13), NAC (9), ERF (9), bHLH (9), and G2-like families (5; **Table S10**). A total of 16 transcription factors were possibly associated with metabolism of linalool (v01), methyl benzoate (v06), leaf acetate (v10), and methyl anthranilate (v26), which were mainly associated with ZF-HD (1), bHLH (2), NAC (2), HSF (1), LBD (1), ERF (1), DBB (1), G2-like (1), and MYB (6; **Figure 6B** and **Table S10**). The heatmap showed that *HF20331*, *HF40059* and *HF38968*, *HF03111*, and *HF21131*, *HF11756* and *HF41805* were closely associated with linalool, methyl benzoate, leaf acetate, and methyl anthranilate, respectively (**Figure 6B**).

## Discussion

At present, the most commonly used methods to collect volatile compounds from plant flowers are as follows: dynamic headspace collection–adsorption, adsorption–solvent desorption, and SPME. By combining collection, extraction, concentration, and sample input, SPME is convenient and fast, which increases the accuracy and sensitivity of the analysis (Matich et al., 1996; Bertoli et al., 2003; Saison et al., 2009a). After the plant volatile substances are collected, they must be separated and analyzed. The current commonly used methods for this are as follows: GC-MS, GC-olfactometry, and electronic nose (Ong and Acree, 1998; Raguso and Pellmyr, 1998; Minh Tu et al., 2002). Analysis by GC-MS to detect volatiles is a relatively mature procedure and is widely used in the analysis of floral volatile components (Bertoli et al., 2003; Risticevic et al., 2010; Zhou et al., 2022). Chemical components were identified by searching a standard atlas database, and the compounds were quantified by ion flow peak area normalization with appropriate standards. This method has high separation efficiency, a wide detection range, and high sensitivity, and thus plays an irreplaceable role in the trace analysis.

### Flower volatile compounds and fragrant characteristics in the apple flowers

The characteristics of floral fragrances are determined by the volatile species and content, and can vary at different flowering stages (Negre et al., 2003; Dotterl et al., 2006). Using the methods of HS-SPME and GC-MS, 56 aroma volatiles were identified at four stages of flowering in the three crabapple materials. Phenylpropane/benzene ring-types compounds were the predominant fragrant volatiles. Using a similar method, 31 volatile substances (predominantly alcohols) were detected in flowers of red ‘Fuji’ apple, of which the most abundant volatiles were benzyl alcohol, *cis*-3-hexene-1-alcohol, linalool, and orange-nitrile (Ma et al., 2018). Solvent extraction and GC-MS were used to identify the alcohol-based substances from flowers of ‘Redgold’ apple, such as *cis*-3-hexene-1-alcohol, benzyl alcohol, 2-phenylethanol, cinnamyl alcohol, and octanol (Omata et al., 1990). Thirty fragrant volatiles were detected in the blooming flowers of ‘Pelingo’ apple, of which the most abundant volatiles were terpenoids and linalool (Fraternale et al., 2014).

The proportion and types of floral volatiles in Mr were different from those reported in previous studies of apple (Omata et al., 1990; Fraternale et al., 2014; Ma et al., 2018); in particular, the content and types of esters were comparatively rich. Notably, the odor thresholds in water of some esters are less than 12.10 ng·g^−1^, such as methyl benzoate (0.52 ng·g^−1^), leaf acetate (12.10 ng·g^−1^), and methyl anthranilate (3.00 ng·g^−1^) (Pino and Quijano, 2012; Zhu et al., 2018). In contrast to the flowers of certain apple cultivars, alcohols were the main fragrant volatiles, but the odor thresholds of some alcohols were higher than 1000 ng·g^−1^, such as benzyl alcohol (10000), (E)-3-hexen-1-ol (1550) and hexyl alcohol (2500) (Zhu et al., 2018; Kang et al., 2019). Therefore, these differences may be an important reason for the strong fragrance of SF flowers.

The application of research results on the volatile substances of plants for insect pollination may reduce manpower and improve the pollination efficiency. Previous studies have shown that floral fragrance provides important signals for attraction of long- and short-distance visits, and floral-scent gradients formed by different plants or different floral organs of the same plant can help to direct pollinators (Fraternale et al., 2014). Approximately 80% of the pollinators of apple are bees, of which 98% are sensitive to various volatile compounds, including (*Z*)-3-hexenyl ester, limonene, and benzaldehyde. Daniele believes that linalool is a typical floral substance that attracts pollinators, and that aromatic esters, such as benzyl acetate, are important components of floral fragrance to attract bees for pollination of orchids (Fraternale et al., 2014). Among the volatile substances identified from flowers of crabapple, the presence of benzaldehyde, benzyl acetate, linalool, and other substances was confirmed, especially in the main period of fragrance emission, and aromatic esters accounted for the highest proportion of aromatic substances. This finding is consistent with the timing of pollination activities of bees in the early stage of flower opening and is beneficial to improve the pollination efficiency of apple.

### Volatile contents and gene expression patterns in the SF flowers

In SF flowers, methyl benzoate, linalool, leaf acetate, and methyl anthranilate were the key and predominant fragrant volatiles. These substances produce a rich and distinctive fragrance and provide a strong sensory experience. This not only benefits from the combination of volatile content and odor threshold, but also includes the synergistic effects among fragrant volatiles (Miyazawa et al., 2008; Zhu et al., 2017). Similarly, the contents of some floral volatiles might be associated with biosynthesis and metabolism pathways. Functional and regulatory genes play a crucial role in volatile synthesis and metabolism. Recent application of omics studies, such as metabolome, transcriptome, and methylome analyses, have improved the understanding of the production of fragrant volatiles (Han et al., 2022; Zhou et al., 2022). From function annotation of the DEGs, terpene synthase, oxidoreductase activity, *O*-methyltransferase activity, and phenylpropanoid biosynthesis were significantly enriched in the comparison of SF with WF or Mr. These results provide insights to clarify the strong fragrance production in SF, but additional studies of the gene functions associated with the key and predominant fragrant volatiles are required.

Most floral volatiles in plants belong to three main types comprising terpene compounds, fatty acid derivatives, and phenylpropane/benzene ring-type compounds (Pichersky and Dudareva, 2007; Rodriguez-Concepcion and Boronat, 2002; Muhlemann et al., 2014; Li et al., 2018). Terpenes are ubiquitous in plant vegetative and flower organs, and are the largest class of naturally occurring substances in flowering plants. Terpenes are synthesized by two pathways: the mevalonate pathway and the methylerythritol phosphate pathway (Nagegowda, 2010; Pulido et al., 2012; Abbas et al., 2017). As one common monoterpenoid in floral volatiles, methylerythritol phosphate is the main pathway for linalool synthesis, in which 1-deoxy-D-xylulose-5-phosphate synthase and 1-deoxyxylulose-5-phosphate reductoisomerase are the rate-limiting enzymes and thus play important roles in the regulation of linalool synthesis. Terpene synthase genes (TPS) play roles in linalool synthesis, such as in *Arabidopsis thaliana* and *Osmanthus fragrans* (Han et al., 2022). Fatty acid derivatives, including saturated and unsaturated short-chain alcohols, aldehydes, and esters, are the second largest class of floral fragrant volatiles. Lipoxygenase (LOX), hydroperoxide lyase (HDL), alcohol dehydrogenase (ADH), and alcohol acyltransferase (AAT) are crucial enzymes in the metabolic pathway of fatty acid derivatives, and are involved in volatiles formation in many fruit (Schwab et al., 2008; Dudareva et al., 2013; Muhlemann et al., 2014; Wang et al., 2022). However, the expression patterns of homologous genes associated with linalool synthesis were inconsistent with the linalool content in SF, WF, and Mr. The shikimic acid pathway is the initial origin of phenylpropanes, starting from phenylalanine, and the first committed enzyme is phenylalanine ammonia-lyase. In this pathway, phenylalanine aminotransferase, shikimate kinase, and AS are responsible for methyl anthranilate synthesis (Pichersky and Gershenzon, 2002; Muhlemann et al., 2014; Vogt, 2010). At present, certain genes involved in the aforementioned synthetic pathways can be isolated and identified, but their expression patterns and regulatory mechanisms require further study.

## Data availability statement

The data presented in the study are deposited in the NCBI GEO repository, accession number GSE216719 (https://www.ncbi.nlm.nih.gov/geo/query/acc.cgi?acc=GSE216719).

## Author contributions

GL, JL, HZ and JS participated in the experimental design and data analysis. GL, JL, YL, SZ, PW, and JL performed material sampling, field measurements and the laboratory data measurement. GL, JL, HZ, MT and JS participated in the paper writing and manuscript amending. All authors contributed to the article and approved the submitted version.
