# Supplementary material for: Volatile metabolome and floral transcriptome analyses reveal the volatile components of strongly fragrant progeny of Malus × robusta
Source: Front Plant Sci. 2023 Jan 20;14:1065219. doi: 10.3389/fpls.2023.1065219 (PMC9895795; doi:10.3389/fpls.2023.1065219)
Supplement: Supplementary file 1 [file DataSheet_1.docx]

**Figure S1 Flower phenotypes in different floral organs at ES.**

The a, b, c, and d represent petals, pistil, stamens, and calyx and bracts. Scale bars =8.0 mm.

**
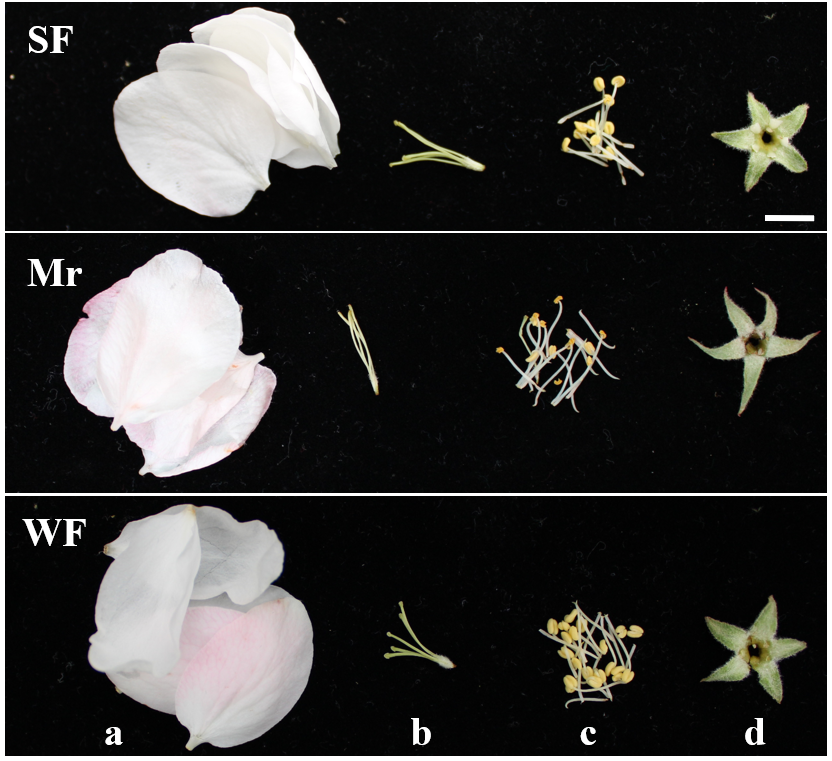
**

**Table S1 Volatile compounds of the three crabapple flowers**

| **Volatile**  **Name** | **Retention Time (min)** | **Volatile Code** | | **Molecular Formula** | **CAS Number** | |
| --- | --- | --- | --- | --- | --- | --- |
| Linalool | 15.82 | v01 | | C_10_H_18_O | 78-70-6 | |
| Methyl octanoate | 16.65 | v02 | | C_9_H_18_O_2_ | 111-11-5 | |
| Methyl laurate | 27.99 | v03 | | C_13_H_26_O_2_ | 111-82-0 | |
| Methyl hexadecanoate | 36.78 | v04 | | C_17_H_34_O_2_ | 112-39-0 | |
| Linalyl acetate | 15.84 | v05 | | C_12_H_20_O_2_ | 115-95-7 | |
| Methyl benzoate | 15.70 | v06 | | C_8_H_8_O_2_ | 93-58-3 | |
| (3E)-3-Hexen-1-ol | 7.12 | v07 | | C_6_H_12_O | 928-97-2 | |
| Decane | 12.17 | v08 | | C_10_H_22_ | 124-18-5 | |
| Methyl tetradecanoate | 32.61 | v09 | | C_15_H_30_O_2_ | 124-10-7 | |
| Leaf acetate | 12.44 | v10 | | C_8_H_14_O_2_ | 3681-71-8 | |
| Heneicosane | 40.09 | v11 | | C_21_H_44_ | 629-94-7 | |
| Naphthalene | 18.72 | v12 | | C_10_H_8_ | 91-20-3 | |
| 1,3-Dimethylbenzene | 7.57 | v13 | | C_8_H_10_ | 108-38-3 | |
| Tetradecane | 24.79 | v14 | | C_14_H_30_ | 629-59-4 | |
| 3-Carene | 14.01 | v15 | | C_10_H_16_ | 13466-78-9 | |
| Methyl 2-methylbutyrate | 5.03 | v16 | | C_6_H_12_O_2_ | 868-57-5 | |
| Nonadecane | 36.25 | v17 | | C_19_H_40_ | 629-92-5 | |
| 4-Methyl-Decanes | 14.42 | v18 | | C_11_H_24_ | 2847-72-5 | |
| 2-Methylnaphthalene | 22.09 | v19 | | C_11_H_10_ | 91-57-6 | |
| cis-3-Hexenyl formate | 7.12 | v20 | | C_7_H_12_O_2_ | 33467-73-1 | |
| Methyl acetate | 2.69 | v21 | | C_3_H_6_O_2_ | 79-20-9 | |
| Methyl hexanoate | 9.36 | v22 | | C_7_H_14_O_2_ | 106-70-7 | |
| 2-Ethylhexanol | 13.26 | v23 | | C_8_H_18_O | 104-76-7 | |
| Isoamylol | 4.23 | v24 | | C_5_H_12_O | 123-51-3 | |
| Benzyl alcohol | 13.50 | v25 | | C_7_H_8_O | 100-51-6 | |
| Methyl anthranilate | 23.38 | v26 | | C_8_H_9_NO_2_ | 134-20-3 | |
| Hexyl alcohol | 7.46 | v27 | | C_6_H_14_O | 111-27-3 | |
| Benzaldehyde | 10.82 | v28 | | C_7_H_6_O | 100-52-7 | |
| Propylcyclopropane | 7.43 | v29 | | C_6_H_12_ | 2415-72-7 | |
| 2-Methyldecane | 14.53 | v30 | | C_11_H_24_ | 6975-98-0 | |
| 4-Methoxy phenyl oxime | 8.52 | v31 | | C_8_H_9_NO_2_ | 3717-22-4 | |
| Methyl tiglate | 7.43 | v32 | | C_7_H_12_O_2_ | 6622-76-0 | |
| 3-Methyldecane | 14.78 | v33 | | C_11_H_24_ | 13151-34-3 | |
| Linalyl butyrate | 15.83 | v34 | | C_14_H_24_O_2_ | 78-36-4 | |
| Methyl heptenone | 11.72 | v35 | | C_8_H_14_O | 110-93-0 | |
| Decyl aldehyde | 19.31 | v36 | | C_10_H_20_O | 112-31-2 | |
| Linalyl formate | 15.83 | v37 | | C_11_H_18_O_2_ | 115-99-1 | |
| 3-Aminophenylacetylene | 22.07 | v38 | | C_8_H_7_N | 54060-30-9 | |
| Semicarbazide | 2.82 | v39 | | CH_5_N_3_O | 57-56-7 | |
| 1-Phenylpropane-1,2-diol | 13.50 | v40 | | C_9_H_12_O_2_ | 1855-09-0 | |
| Indole | 22.03 | v41 | | C_8_H_7_N | 120-72-9 | |
| Tetracosane | 40.08 | v42 | | C_24_H_50_ | 646-31-1 | |
| 2-N-pentofuran | 11.91 | v43 | | C_9_H_14_O | 3777-69-3 | |
| Cyclohexanol | 7.39 | v44 | | C_6_H_12_O | 108-93-0 | |
| 5-Methyldecane | 14.29 | v45 | | C_11_H_24_ | 13151-35-4 | |
| 1,4-Dimethylbenzene | 7.46 | v46 | | C_8_H_10_ | 106-42-3 | |
| Methyl 2-Ethylhexanoate | 13.81 | v47 | | C_9_H_18_O_2_ | 816-19-3 | |
| Methyl 2-methyl-2-butenoate | 7.44 | v48 | | C_6_H_10_O_2_ | 41725-90-0 | |
| Hexyl acetate | 12.70 | v49 | | C_8_H_16_O_2_ | 142-92-7 | |
| Benzyl acetate | 18.04 | v50 | | C_9_H_10_O_2_ | 140-11-4 | |
| Acethydrazide | 2.60 | v51 | | C_2_H_6_N_2_O | 1068-57-1 | |
| 2-Methylgeng acid | 16.66 | v52 | | C_8_H_16_O_2_ | 1188-02-9 | |
| 2-Methylcyclopentalol | 7.42 | v53 | | C_6_H_12_O | 24070-77-7 | |
| Methyl butyrate | 4.22 | v54 | | C_5_H_10_O_2_ | 623-42-7 | |
| 1,8-Octandiol | 16.01 | v55 | C_8_H_18_O_2_ | | | 629-41-4 |
| 1,3-trans,5-cis-Octatriene | 13.61 | v56 | C_8_H_12_ | | | 40087-61-4 |
| Cinnamyl alcohol | 22.38 | v57 | C_9_H_10_O | | | 104-54-1 |
| Geranylacetone | 26.26 | v58 | C_13_H_22_O | | | 3796-70-1 |
| cis-3-Hexenyl benzoate | 29.25 | v59 | C_13_H_16_O_2_ | | | 25152-85-6 |
| Phenethyl alcohol | 0.31 | v60 | C_8_H_10_O | | | 60-12-8 |
| 3-Phenyl-1-propanol | 20.15 | v61 | C_9_H_12_O | | | 122-97-4 |
| Cinnamaldehyde | 21.42 | v62 | C_9_H_8_O | | | 14371-10-9 |
| Benzyl 2-methyl-2-butenoate | 27.48 | v63 | C_12_H_14_O_2_ | | | 67674-41-3 |
| Pentanoic acid | 24.79 | v64 | C_12_H_16_O_2_ | | | 10361-39-4 |
| Benzyl benzoate | 33.74 | v65 | C_14_H_12_O_2_ | | | 120-51-4 |
| Benzyl butyrate | 23.45 | v66 | C_11_H_14_O_2_ | | | 103-37-7 |
| Ethyl decanoate | 24.69 | IS | C_12_H_24_O_2_ | | | 110-38-3 |

**Table S2 Volatiles of SF flowers in four flowering stages (µg·g^-1^)**

| **Volatile Type** | **Volatile Name** | **Volatile Code** | **EB** | **LB** | **ES** | **LS** |
| --- | --- | --- | --- | --- | --- | --- |
| Fatty acid derivatives | Methyl octanoate | v02 | 42.94 | 90.14 | 128.88 | 126.19 |
|  | Methyl laurate | v03 | 26.97 | 26.73 | 65.82 | 39.66 |
|  | Methyl hexadecanoate | v04 | 26.00 | 62.57 | 80.87 | 36.27 |
|  | (E)-3-Hexen-1-ol | v07 | 12.59 | 112.48 | 394.70 | 394.03 |
|  | Methyl tetradecanoate | v09 | 7.28 | 8.07 | - | - |
|  | Leaf acetate | v10 | 7.12 | 58.62 | 419.96 | 19.46 |
|  | Methyl 2-methylbutyrate | v16 | 2.73 | 14.02 | 48.48 | 36.32 |
|  | Linalyl butyrate | v34 | - | 105.19 | - | - |
|  | cis-3-Hexenyl formate | v29 | - | 32.52 | - | - |
|  | Methyl acetate | v21 | - | 28.23 | 39.19 | 42.30 |
|  | Methyl hexanoate | v22 | - | 27.48 | 56.35 | 22.62 |
|  | 2-Ethylhexanol | v23 | - | 6.09 | - | 17.67 |
|  | Isoamylol | v24 | - | 4.15 | - | 28.64 |
|  | Hexyl alcohol | v27 | - | - | 379.57 | - |
| Terpene compounds | Linalool | v01 | 100.44 | 280.30 | 201.16 | 63.37 |
|  | Linalyl acetate | v05 | 20.21 | - | - | - |
|  | 3-Carene | v15 | 3.18 | 9.86 | - | - |
|  | 1,3-trans,5-cis-Octatriene | v56 | - | 12.31 | - | - |
|  | Methyl tiglate | v32 | - | - | - | 15.48 |
| Phenylpropane or benzene ring compounds | Methyl benzoate | v06 | 14.20 | 426.37 | 692.90 | 400.30 |
|  | Naphthalene | v12 | 7.05 | 8.45 | 128.96 | 15.58 |
|  | 1,3-Dimethylbenzene | v13 | 3.87 | 12.99 | 384.79 | 10.12 |
|  | 2-Methylnaphthalene | v19 | 2.42 | - | - | - |
|  | Benzyl alcohol | v25 | - | - | 668.47 | 458.12 |
|  | Methyl anthranilate | v26 | - | - | 390.20 | - |
|  | Benzaldehyde | v28 | - | - | 193.48 | - |
|  | 4-Methoxy phenyl oxime | v31 | - | - | - | 327.78 |
| Others | Decane | v08 | 8.03 | 197.10 | 233.12 | 337.59 |
|  | Heneicosane | v11 | 7.11 | - | - | - |
|  | Tetradecane | v14 | 3.18 | - | - | - |
|  | Nonadecane | v17 | 2.65 | - | - | - |
|  | 4-Methyl-Decanes | v18 | 2.64 | - | 10.45 | - |
|  | Propylcyclopropane | v29 | - | - | 31.53 | - |
|  | 2-Methyldecane | v30 | - | - | 14.27 | - |
|  | 3-Methyldecane | v33 | - | - | - | 14.27 |

Note: Data represent the means. ‘-’ represents that the substance was not detected.

**Table S3 Volatiles of WF flowers in four flowering stages (µg·g^-1^)**

| **Volatile Type** | **Volatile Name** | **Volatile Code** | **EB** | **LB** | **ES** | **LS** |
| --- | --- | --- | --- | --- | --- | --- |
| Fatty acid derivatives | Methyl octanoate | v02 | 32.77 | 122.67 | 60.30 | 48.47 |
|  | Methyl laurate | v03 | 17.26 | 20.04 | 53.25 | 10.14 |
|  | Methyl hexadecanoate | v04 | 8.78 | 96.56 | 17.78 | - |
|  | (E)-3-Hexen-1-ol | v07 | 6.34 | - | 54.97 | - |
|  | Methyl tetradecanoate | v09 | 8.98 | - | 39.19 | - |
|  | Leaf acetate | v10 | - | 40.31 | - | - |
|  | Methyl 2-methylbutyrate | v16 | - | 1.59 | - | 47.44 |
|  | Methyl acetate | v21 | - | 12.30 | - | 1.30 |
|  | Methyl hexanoate | v22 | - | - | 11.99 | 24.75 |
|  | 2-Ethylhexanol | v23 | - | - | 49.69 | 21.47 |
|  | Linalyl butyrate | v34 | 68.30 | 382.56 | - | - |
|  | Methyl heptenone | v35 | 3.63 | 15.80 | - | 13.77 |
|  | Decyl aldehyde | v36 | 2.48 | - | - | - |
| Terpene compounds | Linalool | v01 | - | - | 40.72 | 46.54 |
|  | Linalyl acetate | v05 | - | - | 68.92 | - |
|  | Methyl tiglate | v32 | - | - | - | 28.72 |
|  | Linalyl formate | v37 | - | 500.38 | - | - |
| Phenylpropane or benzene ring compounds | Methyl benzoate | v06 | 29.86 | 228.53 | 476.46 | 81.73 |
|  | Naphthalene | v12 | 10.51 | 27.63 | 6.19 | 5.51 |
|  | 1,3-Dimethylbenzene | v13 | - | 41.68 | 61.93 | 34.15 |
|  | 2-Methylnaphthalene | v19 | 3.59 | - | - | - |
|  | Benzyl alcohol | v25 | - | - | 243.03 | 54.35 |
|  | Benzaldehyde | v28 | - | - | 218.99 | - |
|  | 1-Phenylpropane-1,2-diol | v40 | - | - | 92.60 | - |
|  | Indole | v41 | - | - | 44.47 | - |
| Others | Decane | v08 | 42.38 | 83.87 | 83.93 | 22.67 |
|  | 2-Methyldecane | v30 | - | - | - | 10.31 |
|  | 3-Aminophenylacetylene | v38 | - | 41.63 | - | - |
|  | Semicarbazide | v39 | - | 18.58 | - | - |
|  | Tetracosane | v42 | - | - | 43.76 | - |
|  | 2-N-pentofuran | v43 | - | - | - | 1.38 |

Note: Data represent the means. ‘-’ represents that the substance was not detected.

**Table S4 Volatiles of Mr flowers in four flowering stages (µg·g^-1^)**

| **Volatile Type** | **Volatile Name** | **Volatile Code** | **EB** | **LB** | **ES** | **LS** |
| --- | --- | --- | --- | --- | --- | --- |
| Fatty acid derivatives | Methyl octanoate | v02 | 38.82 | 27.90 | - | 41.19 |
|  | Methyl laurate | v03 | 16.76 | 34.45 | 21.27 | 20.64 |
|  | (E)-3-Hexen-1-ol | v07 | 121.59 | 112.31 | 154.38 | - |
|  | Leaf acetate | v10 | 117.45 | 127.40 | 217.17 | 217.30 |
|  | Methyl 2-methylbutyrate | v16 | 12.11 | 28.84 | 17.42 | 23.58 |
|  | cis-3-Hexenyl formate | v29 | - | - | 89.10 | 29.55 |
|  | Methyl acetate | v21 | - | 17.20 | - | - |
|  | Methyl hexanoate | v22 | 8.77 | 44.99 | 55.13 | 60.24 |
|  | Methyl heptenone | v35 | 5.66 | 29.14 | 29.03 | 38.68 |
|  | Cyclohexanol | v44 | 95.28 | 109.51 | 64.95 | - |
|  | Methyl 2-Ethylhexanoate | v47 | - | 35.15 | 91.57 | 111.69 |
|  | Methyl 2-methyl-2-butenoate | v48 | - | - | 52.57 | - |
|  | Hexyl acetate | v49 | - | - | 31.31 | 30.51 |
|  | 2-Methylgeng acid | v52 | - | - | 8.09 | - |
|  | Methyl butyrate | v54 | - | - | 3.91 | - |
|  | 1,8-Octandiol | v55 | - | - | - | 14.75 |
| Terpene compounds | Linalool | v01 | 81.96 | 59.68 | 32.12 | 19.64 |
|  | Linalyl acetate | v05 | - | 48.07 | - | - |
| Phenylpropane or benzene ring compounds | Methyl benzoate | v06 | - | - | 411.88 | 380.95 |
|  | Naphthalene | v12 | 4.42 | - | 9.74 | 14.34 |
|  | 1,3-Dimethylbenzene | v13 | 4.45 | 29.33 | 18.54 | 21.45 |
|  | Benzyl alcohol | v25 | - | - | - | 160.22 |
|  | Benzaldehyde | v28 | - | - | 8.51 | - |
|  | 4-Methoxy phenyl oxime | v31 | 112.18 | 80.63 | 78.56 | 92.63 |
|  | 1,4-Dimethylbenzene | v46 | - | 14.00 | - | 11.47 |
|  | Benzyl acetate | v50 | - | - | 31.14 | - |
| Others | Decane | v08 | 92.21 | 111.04 | 73.48 | 183.23 |
|  | Propylcyclopropane | v29 | 17.11 | 30.03 | - | 38.52 |
|  | 2-Methyldecane | v30 | 5.39 | 31.07 | - | - |
|  | 5-Methyldecane | v45 | 4.89 | - | - | - |
|  | Acethydrazide | v51 | - | - | 11.41 | 27.19 |
|  | 2-Methylcyclopentalol | v53 | - | - | 5.24 | - |

Note: Data represent the means. ‘-’ represents that the substance was not detected.

**Table S5 Threshold values of volatile components in flowers**

| **Volatile component** | **Threshold value (ng·g^-1^)** |
| --- | --- |
| Methyl benzoate | 0.52 |
| Methyl hexanoate | 70-84 |
| (E)-3-Hexen-1-ol | 1550 |
| 1,3-Dimethylbenzene | 5500 |
| Linalool | 6 |
| Benzaldehyde | 350-3500 |
| Methyl octanoate | 200 |
| Leaf acetate | 12.1 |
| Methyl hexadecanoate | 4×10^6^ |
| Benzyl alcohol | 10000 |
| Methyl anthranilate | 3 |
| Hexyl alcohol | 2500 |
| Methyl acetate | 5.5×10^5^ |
| Linalyl acetate | 100-1000 |
| 2-Ethylhexanol | 2.7×10^5^ |
| Indole | 140 |
| Cyclohexanol | 10^6^-2×10^6^ |
| Hexyl acetate | 2 |
| Benzyl acetate | 2 |
| Methyl heptenone | 50 |
| Methyl butyrate | 60-76 |

Note: Unlisted volatiles indicated that have not been queried.

**Table S6 Statistics of RNA-seq data**

| **Sample ID** | **Raw reads** | **Clean reads** | **Clean bases (bp)** | **Error rate (%)** | **Q20 (%)** | **Q30 (%)** | **GC content**  **(%)** |
| --- | --- | --- | --- | --- | --- | --- | --- |
| SF_EB_1 | 48859932 | 48276324 | 7.24G | 0.03 | 97.94 | 94.08 | 46.46 |
| SF_EB_2 | 44491616 | 43960034 | 6.59G | 0.03 | 97.84 | 93.85 | 46.48 |
| SF_EB_3 | 42747302 | 42285694 | 6.34G | 0.03 | 97.93 | 94.04 | 46.50 |
| SF_ES_1 | 59419918 | 58720682 | 8.81G | 0.03 | 97.96 | 94.12 | 46.48 |
| SF_ES_2 | 48929334 | 48364236 | 7.25G | 0.02 | 98.04 | 94.27 | 46.49 |
| SF_ES_3 | 46890406 | 46351616 | 6.95G | 0.02 | 98.04 | 94.32 | 46.63 |
| SF_LS_1 | 50406666 | 49796586 | 7.47G | 0.03 | 97.93 | 94.05 | 46.70 |
| SF_LS_2 | 43357440 | 42856546 | 6.43G | 0.02 | 98.03 | 94.29 | 46.79 |
| SF_LS_3 | 45769526 | 45265732 | 6.79G | 0.02 | 98.04 | 94.34 | 46.84 |
| WF_EB_1 | 55521412 | 54742420 | 8.21G | 0.02 | 98.05 | 94.41 | 46.72 |
| WF_EB_2 | 76589500 | 75735776 | 11.36G | 0.03 | 98.01 | 94.24 | 46.72 |
| WF_EB_3 | 60041400 | 59363212 | 8.90G | 0.03 | 97.96 | 94.13 | 46.74 |
| WF_ES_1 | 61290968 | 60703574 | 9.11G | 0.03 | 97.99 | 94.19 | 46.52 |
| WF_ES_2 | 54520338 | 53927266 | 8.09G | 0.03 | 97.76 | 93.67 | 46.56 |
| WF_ES_3 | 46911274 | 46457046 | 6.97G | 0.03 | 97.73 | 93.58 | 46.45 |
| WF_LS_1 | 61695742 | 61045480 | 9.16G | 0.03 | 97.90 | 93.97 | 46.52 |
| WF_LS_2 | 50762648 | 50234262 | 7.54G | 0.03 | 98.01 | 94.21 | 46.57 |
| WF_LS_3 | 52995942 | 52534548 | 7.88G | 0.02 | 98.04 | 94.29 | 46.64 |
| Mr_EB_1 | 63110546 | 62453882 | 9.37G | 0.03 | 97.98 | 94.16 | 46.61 |
| Mr_EB_2 | 52423656 | 51940392 | 7.79G | 0.02 | 98.08 | 94.31 | 46.51 |
| Mr_EB_3 | 50907744 | 50324544 | 7.55G | 0.03 | 97.80 | 93.65 | 46.52 |
| Mr_ES_1 | 50768156 | 50241114 | 7.54G | 0.03 | 97.97 | 94.12 | 46.57 |
| Mr_ES_2 | 45322638 | 44879760 | 6.73G | 0.02 | 98.03 | 94.24 | 46.81 |
| Mr_ES_3 | 50546666 | 50021272 | 7.50G | 0.02 | 98.06 | 94.34 | 46.73 |
| Mr_LS_1 | 51654918 | 51129878 | 7.67G | 0.03 | 97.97 | 94.12 | 46.64 |
| Mr_LS_2 | 50186666 | 49675252 | 7.45G | 0.03 | 97.84 | 93.80 | 46.73 |
| Mr_LS_3 | 45972862 | 45443176 | 6.82G | 0.03 | 97.85 | 93.81 | 46.70 |

Note: Numbers followed the underlines represent the first, second and third biological replicates.

**Table S7 Comparison data of GO and KEGG enrichments**

**Table S8 Homologous genes associated with production of floral volatiles**

**Table S9 PCC and MR values in the connection analyses**

**Table S10 Transcription factors of DEGs in three stages**
